# Supplementary material for: Study on the diffusion and deposition law of pore slurry in gangue filling zone based on CFD-DEM coupling
Source: PLoS One. 2024 Feb 29;19(2):e0297151. doi: 10.1371/journal.pone.0297151 (PMC10903837; doi:10.1371/journal.pone.0297151)
Supplement: Attachment — Submitted filename: Review.docx [file pone.0297151.s001.docx]

**Title:** Study on the Diffusion and Deposition Law of Pore Slurry in Gangue Filling Zone Based on CFD-DEM Coupling

Based on my review of the work, I suggest that the authors address the following observations before it is recommended for publication in PLoS ONE.

**1.   Abstract:** Lines 13, 19, 25 and all through. When writing scientific papers, avoid using personal pronouns such as "we", "us", etc.

**2.   Introduction:** It is recommended to have the work proofread by a native English speaker to ensure that there are no grammatical errors. In line 62 and throughout, insert the reference number immediately after the author's name. It is recommended to use "Gangue" for gangue, and to write the novelty of the work clearly at the end of the introduction.

**3. CFD is not limited to Navier-Stokes equations.** There is no doubt that the Navier–Stokes equation applies to single-phase moving fluid in Darcy-Forchheimer laminar flow regimes. Algebraic yPlus equations, Low Raynolds Average Navier - Stokes equations, Spallart Almaras equations, L-Level equations, and k-e and k-w equations apply to flows outside the laminar region for fast-flowing fluids. Kindly visit Otaru & Samuel 2021 https://iopscience.iop.org/article/10.1088/2053-1591/abf3e2

**4. The continuity equation is defined by Equation 2, which must equal zero.**

**5.** Section 2.2 and 3 seem like reading literature. It needs to be rewritten and reorganized in third person singular. There must be clear details on how the research was conducted in this section.

**6. Explain the procedure for characterizing pore structures, the experimental procedure for analyzing the hydrodynamics of packed structures, the parameters measured, the physics solved, boundary conditions for modeling, mesh structure type, and mesh independence study.**

**7.** How does the tortuosity of these packed structures affect their hydrodynamics?
