# Supplementary material for: Study on the diffusion and deposition law of pore slurry in gangue filling zone based on CFD-DEM coupling
Source: PLoS One. 2024 Feb 29;19(2):e0297151. doi: 10.1371/journal.pone.0297151 (PMC10903837; doi:10.1371/journal.pone.0297151)
Supplement: Attachment — Submitted filename: Respond to reviewers.docx [file pone.0297151.s002.docx]

Dear Reviewers:

Thank you for your letter and for the reviewers’ comments concerning our manuscript entitled “**Study on the Diffusion and Deposition Law of Pore Slurry in Gangue Filling Zone Based on CFD-DEM Coupling**” (ID: **PONE-D-23-29771**). Those comments are all valuable and very helpful for revising and improving our paper, as well as the important guiding significance to our researches. We have studied comments carefully and have made correction which we hope meet with approval. (Changes in the text are marked in red)

Based on my review of the work, I suggest that the authors address the following observations before it is recommended for publication in PLoS ONE.

1. **Abstract:** Lines 13, 19, 25 and all through. When writing scientific papers, avoid using personal pronouns such as "we", "us", etc.

Thanks to your suggestion, we have revised the summary again and removed such as "we", "us", etc.

2. **Introduction:** It is recommended to have the work proofread by a native English speaker to ensure that there are no grammatical errors. In line 62 and throughout, insert the reference number immediately after the author's name. It is recommended to use "Gangue" for gangue, and to write the novelty of the work clearly at the end of the introduction.

**Thanks for your suggestions, we have revised the whole article to modify the sentence, and changed gangue to Gangue.**

3. **CFD is not limited to Navier-Stokes equations.** There is no doubt that the Navier–Stokes equation applies to single-phase moving fluid in Darcy-Forchheimer laminar flow regimes. Algebraic yPlus equations, Low Raynolds Average Navier - Stokes equations, Spallart Almaras equations, L-Level equations, and k-e and k-w equations apply to flows outside the laminar region for fast-flowing fluids. Kindly visit Otaru & Samuel 2021 <https://iopscience.iop.org/article/10.1088/2053-1591/abf3e2>

**Yes, your suggestion is very good. We have studied the Kindly visit Otaru & Samuel 2021 article, which constructed the gaps and channels through CT 3D modeling. Due to the constructed pore channels, the mesh density and accuracy will be improved during the meshing of CFD. And grid independence verification is required in CFD. The core of CFD-DEM coupling is the relationship between particle size and mesh size. In this study, because gangue particles are fixed porous media, the force calculation is not considered, and only the motion and deposition of slurry particles are considered. In this study, a mesh larger than 10 times the volume of particles is adopted and the grid neutrality is verified. References are also cited.**

The viscosity of the slurry tested in this study is 1.0e^-5^ m/s^2^ and the density is 1100 kg/m^3^. The parameters of grout have an important influence on the flow state in the pores, so the law of grout diffusion and particle settlement can be correctly analyzed on the basis of determining the parameters of grout and particle. On the basis of determining the material for numerical simulation, the convergence of the mesh and the relationship between the mesh particles are further analyzed, as shown in the Fig.4.

Fig. 4 Calculation grid of fluid domain and its relation with particles

In Fig. 4, when the fluid mesh decreases from 0.02m to 0.01m, the monitoring of the flow velocity at the same point shows no significant change, indicating convergence under the conditions of 0.02-~0.01m. In addition, considering whether the drag model is satisfied between the fluid mesh and particle size, The size of the skeleton particles is close to or even larger than that of the fluid grid, but because the skeleton particles only occupy the volume of the fluid without calculating the drag force, only the relationship between the size of the grout particles and the fluid grid is required. In the numerical simulation, the size of the grout particles is 3 times lower than that of the fluid grid, so it meets the requirements of coupling calculation.

4. **The continuity equation is defined by Equation 2, which must equal zero.**

**We are very sorry for the display error caused by the formula format error. We have modified it.**

5. Section 2.2 and 3 seem like reading literature. It needs to be rewritten and reorganized in third person singular. There must be clear details on how the research was conducted in this section.

Thanks for your suggestion, we have revised sections 2.2 and 3. The content of this section has been restated.

6. **Explain the procedure for characterizing pore structures, the experimental procedure for analyzing the hydrodynamics of packed structures, the parameters measured, the physics solved, boundary conditions for modeling, mesh structure type, and mesh independence study.**

Thanks to your suggestion, we have included in the corresponding Chapter 3 the experimental procedure for analyzing the fluid dynamics of filled structures, the packing pattern, and the grid problem solved by numerical simulation.

**7.** How does the tortuosity of these packed structures affect their hydrodynamics?

Journal requirements:

Thanks for your suggestion, we will add a flow diagram to illustrate the effect of pore structure on flow.

Fig. 14 The variation of the flow line in the longitudinal and transverse sections

Comments to the Author

Reviewer #1: The manuscript reports the simulation and experimental study of filling process in the gangue pore injection. The effects of various factors including slurry velocity and particle velocity are modelled. The interesting results are the particle and slurry flow dynamics inducedby the grouting process. The experimental data are presented to compare with simulation results. The topic is relevant to many industrial processes, and is of importance. The manuscript is fairly clear with quite a bit of systematic results. I have following comments for the authors toconsider.

Introduction: Up to line 58 on page 2, the manuscript only explains the importance of filling process. It is excessive. The introduction is a bit too general, lack of depth. What are the models in literature? What are the limitations of these models?

Thanks to your suggestion, we have revised the introduction to remove some descriptive paragraphs and highlight the shortcomings of the current study (in terms of pore structure and particle motion deposition law), while highlighting the characteristics of CFD-DEM coupling in terms of structural pore structure and particle flow.

As for the simulation methods in this work: it is not clear how CFD and DEM are coupled. Please provide the details in section 4.1.

Grassman principle ---Grassmann principle

Equation (2) is not complete.

Due to a problem with the editor, it is not complete. We have modified it.

In table 1, gangue –Gangue

Thank you for your suggestion, we have made changes in the table.

Above Figure 5 in line 285: As shown in Fig. 5. ---This is not a sentence.

We have modified the content to make it smooth.

Figure 5: Colors for the labels are not clear. The contrast can be improved to help visualize the particles.

We have modified Figure 5 to highlight the state of particle distribution and diffusion range.

Figure 8: a series of snapshots should be presented so the shell-shape should be clearly visible. At the moment, the shape is labelled by the curve. But the data do not show it clearly.

We are very sorry, because the slurry particles are stained, which will cause the liquid to turn the whole slurry red, so we can only pass the final stable state

Reviewer #2: DEM Coupling: Enhancing Clarity and Simulation Details"

The paper titled "Study on the Diffusion and Deposition Law of Pore Slurry in Gangue Filling Zone Based on CFD-DEM Coupling" provides a compelling integration of CFD and experimental analysis. Although the text is well-composed, several areas require further clarification and elaboration:

1. The abstract lacks sufficient technical details and methodology. A thorough update of the abstract is essential to provide a comprehensive overview of the study.

Thanks to your suggestion, we have revised the summary to condense the core conclusions and quantitative indicators.

2. Clear elucidation is required concerning the number of parameters and their specific impacts within the context of the study. Additionally, addressing uncertainties regarding the fixation of external parameters in the future, backed by relevant literature, is necessary.

Yes, we mainly compare the experimental and numerical simulation parameters to analyze the pore flow of particles in detail through numerical simulation.

3. Please include more detailed information on the DEM, such as particle size and other relevant properties.

In Table 1, we have described the particle parameters, in addition, we added gangue particles and grout particles to illustrate.

4. Consider replacing section 2.2 with a graphical illustration to enhance understanding.

Yes, thanks for your suggestion, we added the coupled calculation flowchart.

In this study, the above governing equations can be described in detail in the coupling flow through Fig. 2.

Fig. 2 Coupling flow chart of slurry in gangue flow

5. Elaborate on the method of particle-particle interaction employed, considering the existing lack of clarity regarding the CFD and DEM methodologies.

The CFD-DEM coupling method can realize the modeling of particle skeleton structure with high precision. At the same time, the particle state of different scales can be studied by calculating the force of micro-particles through drag force. More importantly, the motion trajectory and distribution range of individual particles can be studied. This is the characteristic of this study and the important reason for adopting this method.

6. Omit ambiguous statements like "In CFD, according to the Reynolds number, flows can be classified into laminar, transitional, and turbulent flows."

Yes, thank you for your advice, We delete In the article. "In CFD, according to the Reynolds number, flows can be classified into laminar, transitional, and turbulent flows." and other related statements

7. Provide clear information regarding the Reynolds number in the simulation and the approach used to determine the turbulence model.

Thanks for your suggestion, we have added Reynolds number to the article to judge the state of flow.$\text{Re}_{p}=\rho_{f}d\mathbf{v}/\mu,$

8. Justify the use of the DEM approach in the study, and consider discussing alternative methods, such as DPM, along with a comparative analysis of computational costs.

In the research, DEM method must be adopted to realize the construction of porous media, while DPM cannot realize the model of particle skeleton, so it cannot be adopted. In addition, the interaction between slurry particles and skeleton particles in the process of particle flow should be considered. We use 64 core servers, and the efficiency of particle and grid volume is good in the current situation.

9. Provide a comprehensive description of the fluid properties and specifications to improve clarity.

Thanks to your suggestion, we increase the parameters of the fluid (viscosity and density) to provide a comprehensive description of the fluid's state.

10.Clearly articulate the simulation process for the CFD-DEM coupling, including information on the use of commercial software or custom coding.

In this study, we adopted the open-source program CFDEM to realize the flow of slurry particles in the gangue pore skeleton. The detailed coupling procedure was reworked in Section 2 and the coupling flow diagram was added.

11. Improve the clarity of Figure 4 by providing a concise explanation of its components, and ensure that the figure accurately represents the results of the CFD analysis.

The porosity of the particles is consistent with the porosity taken in the test, and the porosity is calculated by the volume of the water tank occupied by the particles in the experiment. The fluctuation range of the pore distribution of each section monitored in the numerical simulation is close to that of the experiment, so the numerical simulation can meet the requirements.

12. Enhance the readability of Figure 5 and provide a technical discussion to facilitate a better understanding of the results it presents.

Yes, we have modified the clarity of Figure 5 in the paper, which can clearly show the distribution and deposition range of particles under different grouting speeds.

13. Include a detailed section on grid generation and verification, as these aspects are currently absent.

In Section 3, we add a convergence evaluation of mesh and particle scales to demonstrate the effectiveness and convergence of the numerical simulation.

14. Address the issue with the maximum value for slurry velocity in Figure 9 by adjusting the scaling and ensuring clear visibility.

Thanks to your suggestions, we have modified Figure 9 to highlight the range of clarity and maximum values.

15. Improve the legibility of vectors in Figure 10, considering the 3D nature of pore flows and the necessity of representing them in a 2D format. Include quantitative values throughout the manuscript to enhance the technical analysis.

Yes, we have modified Figure 10 to make it clearer to see the flow of vectors through the pores.

In conclusion, this manuscript necessitates textual revisions and an improved presentation of simulation details. Addressing the concerns in the text and providing a clear depiction of the simulation, particularly Figure 13, is crucial. Please consider these recommendations to ensure the clarityand accuracy of your research.

Let's modify Figure 13 to make it fit.

Reviewer #3: Based on my review of the work, I suggest that the authors address the following observations before it is recommended for publication in PLoS ONE. Please refer to the attached document. I strongly suggest the paper be reviewed by a nature English speaker before submitted therevised copy.

We have improved the quality of the full text and polished it.
