# Supplementary material for: Study on the diffusion and deposition law of pore slurry in gangue filling zone based on CFD-DEM coupling
Source: PLoS One. 2024 Feb 29;19(2):e0297151. doi: 10.1371/journal.pone.0297151 (PMC10903837; doi:10.1371/journal.pone.0297151)
Supplement: Attachment — Submitted filename: Respond to reviewers2.docx [file pone.0297151.s003.docx]

Dear Reviewers:

Thank you for your letter and for the reviewers’ comments concerning our manuscript entitled “**Study on the Diffusion and Deposition Law of Pore Slurry in Gangue Filling Zone Based on CFD-DEM Coupling**” (ID: **PONE-D-23-29771**). Those comments are all valuable and very helpful for revising and improving our paper, as well as the important guiding significance to our researches. We have studied comments carefully and have made correction which we hope meet with approval. (Changes in the text are marked in red)

Journal Requirements:

Please review your reference list to ensure that it is complete and correct. If you have cited papers that have been retracted, please include the rationale for doing so in the manuscript text, or remove these references and replace them with relevant current references. Any changes to thereference list should be mentioned in the rebuttal letter that accompanies your revised manuscript. If you need to cite a retracted article, indicate the article’s retracted status in the References list and also include a citation and full reference for the retraction notice.

Thank you for your suggestion. As the reviewer suggested deleting some descriptive statements, some references supported the descriptive statements. Therefore, after deleting these related statements, we cannot still place the references in that position. Therefore, we have removed the relevant excess references.

Reviewer #2: Thank you for the revised version of the manuscript. While efforts to enhance quality and address previous concerns are evident, several key issues still persist. My feedback on the current version is as follows:

Figure 1's Effectiveness: I am uncertain about the usefulness of Figure 1 in aiding the reader's understanding. The absence of scale, color details, and dimensions limits its effectiveness. I suggest replacing it with a more informative figure.

We have adopted a different version of Figure 1 with added scales. However, due to system issues, we uploaded it in the form of an attachment. The gangue skeleton model in Figure 1 mainly displays the pore structure filled with particles.

Figure 1 Gangue skeleton model

Enhancements for Figure 2: I appreciate Figure 2, but it would benefit from additional details and descriptions to better convey its intended message.

Thank you for your suggestion. In the previous revision, other reviewers suggested using a computational flowchart instead of a statement description. Therefore, we have added a computational flowchart. We will add a brief statement description on top of the flowchart.

The entire calculation process can be expressed as setting the initial velocity of the fluid in CFD simulation, solving the Navier Stokes equation, and using CFD method to simulate fluid motion. Construct the pore structure of gangue particles in DEM simulation. Coupling CFD results with DEM results. At each time step, the drag force received by particles is calculated based on the fluid velocity field to update their motion state. Iterative loop until the predetermined calculation time or convergence standard is reached.

Concerns about Figure 3: The lack of scale in Figure 3 is problematic, as it features an image of an unknown material. This omission is surprising and diminishes the figure's clarity. Also, the relationship between Figure 3a and 3b is unclear and needs explanation.

Fig.3 Measurement of the stacking angle of slurry particles

Thank you for your suggestion. We have added an angle diagram in Figure 3 to illustrate that the numerical simulation and experimental results are consistent. Figure 3 shows the parameter calibration of the experimental and numerical simulation of the stacking angle of rest. When the angle of rest obtained in the experiment is consistent with the simulation results, the parameter calibration is completed. We have tested and found that α Related to β It is consistent, indicating that the parameters of the numerical simulation have been validated.

Clarification Needed for Figure 4: The purpose of Figure 4 is not apparent. It raises several questions, particularly regarding the dark points in the figure. Despite the minimum particle size being 0.008m, the grid size is noted as 0.01m. Can you clarify what is meant by 'satisfying therelationship' in this context?

Fig. 4 Calculation grid of fluid domain and its relation with particles

Thank you for your suggestion. All of our theories are based on the CFD-DEM coupling theory. In unresolved CFD-DEM coupling, the volume of moving particles should be less than 10 times that of the fluid mesh to ensure the effectiveness of the drag force during calculation. Therefore, Figure 4 shows the relationship between moving particles and skeleton particles in the process of grid independence verification. The mesh we used meets the requirements of grid independence, And it conforms to the grid size and particle size relationship of drag force in CFD-DEM coupling calculation, which can be reflected in Figure 4.

Reviewer #3: Most of my comments/observations have been addressed by the authors, but the work still needs to be corrected before it can be published. In my earlier submission, I emphasized that authors should avoid using personal pronouns in their research writing. This area is still notfully addressed. In the Abstract section, for example, "In the plane flow field, we observe"; remove "we" and re-write it as..."In the plane flow field, it was observed that......". Check all through the work as well.

Thanks to your suggestions, we have revised the language issues mentioned in the article, especially the person. All changes have been highlighted in red.
