# Supplementary material for: Study on the diffusion and deposition law of pore slurry in gangue filling zone based on CFD-DEM coupling
Source: PLoS One. 2024 Feb 29;19(2):e0297151. doi: 10.1371/journal.pone.0297151 (PMC10903837; doi:10.1371/journal.pone.0297151)
Supplement: Attachment — Submitted filename: Respond to reviewers2.docx [file pone.0297151.s004.docx]

Dear Editor:

Thank you for your letter and for the reviewers’ comments concerning our manuscript entitled “**Study on the Diffusion and Deposition Law of Pore Slurry in Gangue Filling Zone Based on CFD-DEM Coupling**” (ID: **PONE-D-23-29771**). Those comments are all valuable and very helpful for revising and improving our paper, as well as the important guiding significance to our researches. We have studied comments carefully and have made correction which we hope meet with approval. (Changes in the text are marked in red)

Journal Requirements:

Please review your reference list to ensure that it is complete and correct. If you have cited papers that have been retracted, please include the rationale for doing so in the manuscript text, or remove these references and replace them with relevant current references. Any changes to the reference list should be mentioned in the rebuttal letter that accompanies your revised manuscript. If you need to cite a retracted article, indicate the article’s retracted status in the References list and also include a citation and full reference for the retraction notice.

Respond：Thanks for your suggestion, we have revised the reference. Based on the revised draft, we have placed the reference removed before in the corresponding place, and added our research value as the reference [32], which has been fully disclosed, in addition to adding a reference [7].

[32] wu, Boqiang (2023), “Slurry velocity and particle velocity”, Mendeley Data, V1, doi: 10.17632/6z2wfrw4sm.1

[7] A.J. Otaru, M.B. Samuel, Pore-level CFD investigation of velocity and pressure dispositions in microcellular structures, Materials Research Express. 8 (2021). https://doi.org/10.1088/2053-1591/abf3e2.

Additional Editor Comments:

The authors are suggested to provide further details of the angle of repose i.e. importance of this angle in their study and its numerical value in simulations and experiments (α and β). Also explain how various parameters (Poisson's ratio, Young Modulus and other coefficients) in Table 1 were determined through angle of repose test.

Respond：Thank you for your suggestion. We will provide the following answer to this question. In this study, a combination of numerical simulation and experimental methods was used to test the angle of rest. The density and size of particles can be obtained in the experiment, but it is difficult to obtain the Poisson's ratio, friction coefficient, and recovery coefficient of particles. Therefore, a static angle test is adopted, which measures the filling angle when the particles are filled, and then conducts multiple numerical simulations, changing the parameters of the particles in the numerical simulations to measure the filling static angle. When the static angle of the packing is close to the static angle of the packing, it can be considered that the parameter calibration is completed. This method is commonly used in parameter calibration of discrete element simulation.
